# Regional body composition in college-aged Caucasians from anthropometric measures

**DOI:** 10.1186/1743-7075-4-29

**Published:** 2007-12-30

**Authors:** Cameron B Ritchie, Robert T Davidson

**Affiliations:** 1 Nutrition, Dietetics, & Food Sciences Department, Brigham Young University, Provo, UT, USA

## Abstract

**Background:**

Quantitating fat and lean tissue in isolated body regions may be helpful or required in obesity and health-outcomes research. However, current methods of regional body composition measurement require specialized, expensive equipment such as that used in computed tomography or dual energy x-ray absorptiometry (DEXA). Simple body size or circumference measurement relationships to body composition have been developed but are limited to whole-body applications. We investigated relationships between body size measurements and regional body composition.

**Methods:**

Using DEXA technology we determined the fat and lean tissue composition for six regions of the body in predominantly Caucasian, college-aged men (n = 32) and women (n = 67). Circumference measurements as well as body weight and height were taken for each individual. Equations relating body measurements to a respective regional fat and lean mass were developed using multiple regression analysis.

**Results:**

Multiple regression R^2 ^values ranged from 0.4451 to 0.8953 and 0.1697 to 0.7039 for regional fat and lean mass relationships to body measurements, respectively.

**Conclusion:**

The equations developed in this study offer a simple way of estimating regional body composition in a college-aged adult population. The parameters used in the equations are common body measurements that can be obtained with the use of a measuring tape and weight scale.

## Background

Obesity has been described as an epidemic in many places throughout the world and its prevalence is of great concern. The World Health Organization (WHO) has defined obesity as having a body mass index (BMI) over 30 (body mass/ht^2^). BMI does not directly assess how much fat a person has but is an indirect assessment that assumes that a higher body mass is due to an increasing percentage of the body's mass being fat. Because BMI does not represent true fat content of the body, fat mass index (FMI: fat mass/ht^2^) or percent body fat (%BF) is often used in obesity research. In order to calculate FMI or %BF the true whole-body fat mass must be known.

There are several methods used to assess whole-body fat including: computed tomography (CT), dual-energy x-ray absorptiometry (DEXA), hydrostatic weighing, bioelectrical impedance (BIA), skinfold thickness, and air-displacement plethysmography. Of these, CT and DEXA are also capable of assessing regional body composition which is useful when investigating regional fat changes [1,2] or relating regional fat deposits to disease risk [3-13]. However, access to either CT or DEXA technologies can be prohibitively costly or otherwise inaccessible. In these cases it would be useful to be able to convert simple anthropometric measures into regional fat mass estimates.

In this study, we developed regional body composition prediction equations that used a combination of anthropometric measurements including circumferences, height, weight, and BMI that correlated to regional body composition data obtained using DEXA.

## Methods

### Human Subjects

Ninety-nine predominantly Caucasian college aged volunteers (67 women and 32 men). These subjects volunteered from Brigham Young University where they were recruited mostly from a beginning nutrition class and posted fliers. This study was approved by Brigham Young University's Institutional Review Board and all volunteers gave informed consent.

### Anthropometric Measures

**Figure 1:**
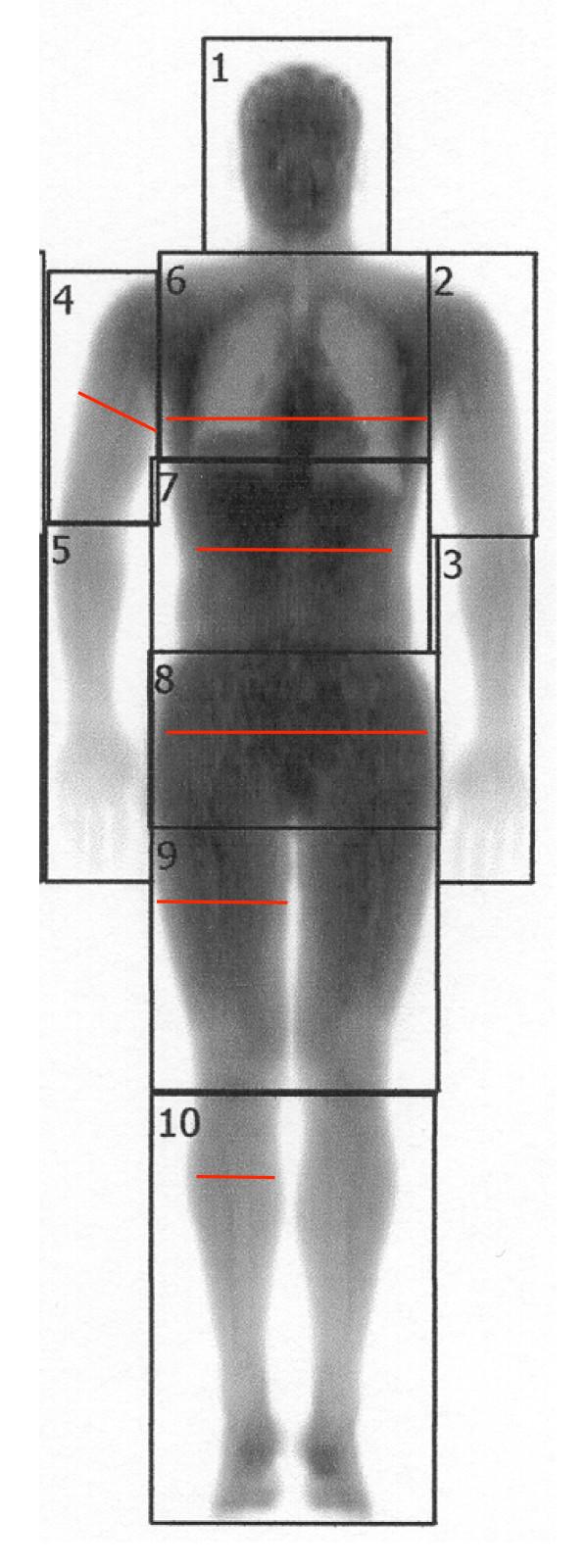
**DEXA scan showing body regions**. Head, chest, upper arm, waist, hips, upper leg, and lower leg regions. Red lines indicate where circumference measurements were taken.

Body height was determined in bare feet using a wall-mounted stadiometer (Perspective Enterprises, Portage, MI). Body weight was measured using a TANTITA TBF-310 scale (Arlington Heights, IL). Body circumference measurements were taken by a trained technician in exact duplicate using a spring-tensioned measuring tape at the following sites: upper arm (at right arm mid-bicep), chest (across fullest measurement of bust), waist (at navel), hips (at fullest measurement including buttocks), upper legs (at right leg mid-thigh), and lower legs (at fullest measurement of right gastrocnemius) (Figure 1).

Regional body composition was determined using LUNAR Dual Energy X-ray Absorptiometry (DEXA) instrument (GE Medical Systems, Madison, WI). The measured amount of lean and fat tissue in each of eight regions was determined including: head, upper arms, lower arms, chest, abdomen, hips, upper leg, and lower leg (Figure 1). Analyses of the arm regions (regions 2 and 4) were combined into one region providing fat and lean mass for both arms.

### Equation Development

The multiple regression equations relating anthropometric measures to fat or lean mass were developed using the statistics software InStat 3 (GraphPad Software, Inc., San Diego, CA). Parameters tested for best fit using multiple regression analysis included: circumference (cm), weight (kg), height (m), body mass index (BMI), and grams of fat associated with the body region.

## Results

### Subjects

The subjects used in this study were predominately college aged Caucasian individuals. They were from all parts of the United States of America. The average subject personal information and measurements taken by the DEXA machine are listed in Table 1. These include the subject's age, height (cm), weight (kg), percent body fat according to DEXA and BIA, fat mass (FM), lean body mass (LBM), and body mass index (BMI). The standard deviations of the mean are listed next to the averages in the table. The ranges from low to high values are listed beneath the average and standard deviation.

**Table 1:** Male and female averages of personal information and measurements

	**Age**	**Mass (kg)**	**Height (m)**	**DEXA % Body Fat**	**FM (g)**	**LBM (g)**	**BMI**
**MALE AVERAGES**	24.3 ± 2.88 (19–33)	82.2 ± 11.39 (64.4–115.5)	1.80 ± 0.06 (1.6–1.9)	20.1 ± 8.02 (4.3–33.0)	16,493.9 ± 8,366.8 (2960–37014)	62,291.7 ± 5,419.3 (53785–75146)	25.5 ± 3.79 (19.2–37.2)
**FEMALE AVERAGES**	23.0 ± 6.21 (18–52)	63.8 ± 11.95 (43.0–104.4)	1.66 ± 0.07 (1.5–1.8)	32.0 ± 7.13 (18.2–51.8)	20,206.0 ± 8,019.9 (7807–44557)	41,615.1 ± 6,447.9 (30287–65965)	23.0 ± 3.62 (17.8–36.5)

### Multiple Regression Analysis

The parameters providing the best correlation (R^2^) and significance were circumference, BMI, weight (kg), and height (m). The multiple regression equations containing these parameters are shown in Tables 2 and 3. The adjusted R^2 ^values and the *p*-values associated with the parameters are also listed in these tables. The standard deviation of the residuals for each equation is listed on Tables 1 and 2.

**Table 2:** Fat regional prediction equations

**Body Region**	**Regional fat mass (g) prediction equation (A: Circumference; B: BMI, C: Weight; D: Height)**	**Multiple Regression Adjusted R^2^**	**Standard Error of Residual (g)**	**Multiple Regression *p*-value**
**Male**				
chest	y = -9754.4 + 56.413*A + 233.61*B	0.8433	211.8	<0.0001
waist	y = -15545 + 100.23*A + 420.94*B	0.8510	388.3	<0.0001
hips	y = -17395 + 160.97*A + 190.77*B	0.7606	446.8	<0.0001
upper leg	y = -8108.9 + 120.21*A + 49.267*C	0.7261	366.0	<0.0001
calf	y = -5809.4 + 127.18*A + 1321.9*D	0.4451	308.7	<0.0001
upper arm	y = -4649.7 + 122.64*A + 949.59*D	0.6073	199.1	<0.0001
**Female**				
chest	y = -14403 + 155.90*A + 134.12*B	0.8286	354.4	<0.0001
waist	y = -7716.2 + 69.439*A + 235.28*B	0.8041	341.4	<0.0001
hips	y = -13285 + 132.63*A + 221.32*B	0.8245	337.8	<0.0001
upper leg	y = -6154.4 + 123.69*A + 48.254*C	0.7338	363.9	<0.0001
calf	y = -4956.0 + 162.73*A + 13.875*C	0.6296	281.7	<0.0001
upper arm	y = -2229.5 + 100.97*A + 13.001*C	0.8953	52.2	<0.0001

**Table 3:** Lean regional prediction equations

**Body Region**	**Regional lean mass (g) prediction equation (A: Circumference; B: Height; C: Weight)**	**Multiple Regression Adjusted R^2^**	**Standard Error of Residual (g)**	**Multiple Regression *p*-value**
**Male**				
chest	y = 3243.0 + 99.751*A - 1114.6*B	0.3861	712.2	0.0003
waist	y = -11795 + 124.63*A + 7283.2*B	0.4911	838.4	< 0.0001
hips	y = -1428.3 -126.72A + 8115.4B + 104.16C	0.4096	740.43	0.0005
upper leg	y = 9875.5 - 156.43*A + 126.06*C	0.4391	700.5	< 0.0001
calf	y = -12279 + 198.10*A + 6384.4*B	0.5746	375.3	< 0.0001
upper arm	y = -8779.1 + 93.336*A + 5455.3*B	0.3253	431.6	0.0013
**Female**				
chest	y = -16125 + 119.35*A + 8425.8*B	0.7039	473.0	< 0.0001
waist	y = 6332.4 - 59.780*A + 80.671*C	0.1697	938.9	0.0010
hips	y = -15180 + 105.97*A + 7261.9*B	0.5790	602.4	< 0.0001
upper leg	y = -14293 + 51.078*A + 10656*B	0.5007	569.8	< 0.0001
calf	y = -8211.5 + 99.987*A + 5590.0*B	0.6606	228.8	< 0.0001
upper arm	y = -3453.0 + 56.120*A + 2469.3*B	0.4451	225.1	< 0.0001

## Discussion

The purpose of this study was to generate equations predicting regional body composition from anthropometric measurements. Anthropometric equations have been developed for predicting whole body fat [14,15], but not regional body fat. Utilizing DEXA technology, we divided the human body into eight regions (Figure 1) and correlated these regional fat and lean tissue masses with several anthropometric measures as shown in Tables 2 and 3.

### Choice of Parameters

The parameters used to predict the regional grams of fat mass with the highest correlation and significance were circumference, BMI, weight (kg), and height (m). The parameters used to predict the regional grams of lean mass with the highest correlation and significance were circumference, weight (kg), and height (m). Each prediction equation used a combination of at least two different parameters in predicting regional body composition. Other parameters tested (ie. FMI, lean body mass index, non-regional circumferences, etc.) were either not significant or provided a lower adjusted R^2 ^value than those listed and are not shown.

### Correlations of fat and lean tissues mass

In our study, regional fat mass prediction equations had higher correlations than regional lean mass equations. Lower correlations in lean mass were likely due to the wide variation in individual muscle mass as a result of resistance training or genetic variation in growth hormone. Resistance training histories were not recorded for this study but would be expected to vary widely between individuals.

### Errors and Limitations of Equations

The standard error of the estimates (or standard deviation of the residuals) are listed in Tables 2 and 3. The error values are smaller than the expected regional fat or lean mass changes during a reasonable weight change. For example, using regional body composition data for 20 – 29 yr-old females from Kotani *et al*. [16], and following the observation of Garrow [17] that the regional percentage contribution to total body fat is similar before and after weight change, and assuming that 75% of weight change is due to fat [18], the change in regional fat mass during a reasonable weight loss of 16.6 kg where BMI changes from 35 to 29 (obese to overweight) for chest, waist, thigh, calf and upper arm is: -1780 g, -5986 g, -2613 g, -814 g and -769 g, respectively, compared to the standard deviation of the residuals for the same regions: 354 g, 341 g, 364 g, 281 g, and 52 g, respectively. This suggests that the errors inherent in the equations reported in this study are much lower than the actual changes in region body composition in this example.

A limit of applicability for the equations reported in this study would be exceeded with a much smaller weight change. A 3 kg wt loss applied to the 20 – 29 yr-old female data from Kotani *et al*. [16] yields changes in regional fat mass of -322 g, -1082 g, -472 g, -147, and -139 g for chest, waist, thigh, calf and upper arm, respectively. In this case, the standard deviation of the residuals for the chest and calf equations reported in this study are slightly larger than the chest and calf regional fat changes from the Kotani data. This suggests that these equations might not be suitable for identifying changes in regional body composition for total body weight losses of 3 kg or less for the Kotani data set. Limits for other data sets might be expected to be different depending on beginning regional fat and lean compositions and BMI.

**Figure 2:**
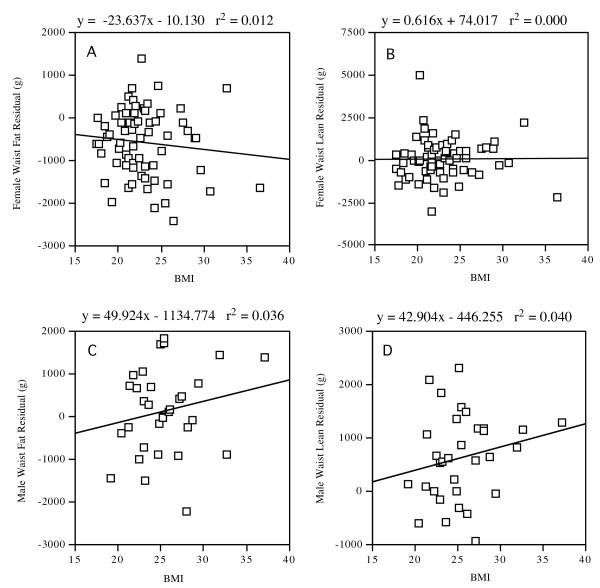
**Waist regression residuals vs. BMI plots**. Multiple regression residuals are plotted against BMI for A) Female waist fat, B) Female waist lean, C) Male waist fat, and D) Male waist lean. Linear regression line, equation and R^2 ^value are shown for each plot.

As lean mass decreases and fat mass regional patterns shift with increasing age [16] these equations would be most adequate for young adults (<35 yrs) with a BMI ranging from normal to obese. BMI for subjects in this study ranged from 18 to 37 and plots of BMI vs. residuals (actual – calculated regional fat or lean mass) showed no skewing at higher BMI values (Figure 2) as BMI or body weight and height were regression parameters for many of the equations.

### Potential Uses and Relevance

Muller *et al. *[1] suggested that regional lean body mass (LBM) is a determinant of resting energy expenditure (REE). As whole body LBM increases more lean mass accumulates in the arm and leg regions than the trunk regions. The ratio of LBM (trunk)/LBM (arms and legs) then decreases and might be used to determine increases in REE.

Okura *et al. *[10] observed in obese women who were on a weight reduction diet that fat tissue in legs had a negative association for some CHD risk factors. They confirmed what had been stated in previous studies that fat tissue in legs was associated with a protective effect against metabolic disorders. However, truncal fat tissue correlated positively with some CHD risk factors at baseline and in response to weight reduction. Central fat and truncal fat deposition, especially intra-abdominal fat tissue, plainly correlated with hypertension and the glucose and lipid metabolic abnormalities. Regional body composition is also important for evaluating the improvement of coronary heart disease (CHD) risk factors during weight-reduction treatment for obesity [10].

Several cross-sectional studies have suggested a relationship connecting regional body composition, especially truncal adiposity measured by DEXA, and many coronary heart disease (CHD) risk factors [4-7,9,10,12,13]. Ito *et al*. [12] suggested that indices for fat distribution such as waist-to-hip ratio and FM_trunk_/FM_legs _were related to cardiovascular risk factors more accurately than overall adiposity. Anthropometric and DEXA indices were comparable in their accuracy of detecting risk factors [10].

### Weight Change

The focus of some obesity research has been to spot reduce fat in different areas of the body [19]. Spot reducing has been largely ineffective except for the reduction of intra-abdominal fat with aerobic exercise [20]. Having a simple method of determining regional body composition may be beneficial in assessing regional body compositional changes while facilitating larger, easier studies or the development and use of at-home body composition-tracking computer software.

## Conclusion

The multiple regression equations produced in this study offer a simple and effective way of estimating regional body composition for individuals in a young adult population. The parameters used in the equations are common anthropometric measurements that can be obtained with the use of a measuring tape and scales.

## List of abbreviations

DEXA-dual-energy X-ray absorptiometry;

Kg-kilograms;

M-meters; 

CHD: Coronary heart disease;

LBM: Lean body mass; 

REE: Resting energy expenditure;

FM: Fat mass;

BMI: Body mass index;

BIA: Bioelectrical impedance analysis;

CT: Computed tomography.

## Competing interests

The author(s) declare that they have no competing interests.

## Authors' contributions

RTD: Study idea and design, IRB approval, data collection, and manuscript editing.

CRB: Data analysis and manuscript writing.
